# Developments in diagnosis and treatment of early hypopharyngeal carcinoma and precancerous lesions: A review

**DOI:** 10.1097/MD.0000000000045617

**Published:** 2025-11-07

**Authors:** Jinhao Cheng, Yuzhi Liu, Xiaowei Tang, Rui Zhao, Yu Bao

**Affiliations:** a Department of Gastroenterology, The Affiliated Hospital of Southwest Medical University, Luzhou, China; b Department of Endoscopy, Sichuan Clinical Research Center for Cancer, Sichuan Cancer Hospital & Institute, Sichuan Cancer Center, Affiliated Cancer Hospital of University of Electronic Science and Technology of China, Chengdu, China; c Department of Gastroenterology and Hepatology, West China Hospital, Sichuan University, Chengdu, China.

**Keywords:** artificial intelligence, conservation surgery, endoscopic mucosal resection, hypopharyngeal cancer, hypopharyngeal carcinoma, narrow-band imaging

## Abstract

Hypopharyngeal carcinoma (HC) is one of the rarest and worst head and neck cancers (HNC) with insidious location, atypical early symptoms, and frequently diagnosed in advanced stages. The risk factors of HC mainly include smoking and drinking. Preventive interventions directed towards environmental risk factors are highly likely to yield far-reaching beneficial effects in reducing the incidence of this disease. The majority of advanced HC patients undergo multimodal treatments that encompass radiotherapy, chemotherapy and surgery, with poor oncological and functional outcomes. The early diagnosis and management of HC are vital for improving the prognosis of patients. Currently, there are few reviews specifically focusing on early HC, thus this review aims to summarize the current advancements in the diagnosis and management of early HC. Magnifying endoscopy with narrow-band imaging (ME-NBI) can improve the detection rate of early lesions and assess the invasion depth by enhancing the visualization of microvascular patterns. In recent years, artificial intelligence (AI) presents a promising prospect in the detection of early HC through deep learning algorithms. Larynx-preserving surgery or radiotherapy alone is predominantly recommended for individuals diagnosed at an early stage, aiming to achieve optimal oncological control while preserving crucial functions. With the increasing detection rates of early HC and the development of surgical equipment, transoral minimally invasive surgery is becoming more widely applied in HC. Its minimally invasive nature offers reduced morbidity and faster recovery, making it an attractive option for eligible patients with early HC.

## 1. Introduction

Hypopharyngeal carcinoma (HC) accounts for about 3% to 5% of head and neck cancers (HNC) with the global incidence exhibiting significant geographic and gender disparities.^[1,2]^ HC manifests a higher incidence in males and is prevalent in regions of high alcohol and tobacco consumption.^[3]^ The etiology of the disease is closely linked to lifestyle factors, with occurrences associated with long-term heavy smoking and alcohol consumption, malnutrition, and concomitant diseases. Patients with HC lack specific symptoms and have a tendency to submucosal dissemination and the encroachment on adjacent cervical organs such as the esophagus and larynx in the early stage.^[4]^ Local lymph node metastasis (LNM) and distant metastasis are prone to occur in the early stage, with the frequent metastasis sites being the lungs, liver, and bones. It is frequently observed that 5-year overall survival (OS) rates for HC patients range from 25% to 35%.^[3]^ Patients with early lesions undergoing surgery or radiation therapy can achieve a 5-year OS rate of approximately 60%.^[5–7]^ Therefore, early diagnosis and management of the disease are of significant clinical value.

Owing to the concealed position of the hypopharynx, traditional imaging examinations have limited capabilities in detecting early HC. The application of magnifying endoscope (ME) and narrow-band imaging (NBI) has made the identification of early stage HC feasible.^[8]^ For the treatment of early HC, both function-preserving surgery and exclusive radiotherapy can achieve satisfactory oncological outcomes and functional preservation.^[9–12]^ Currently, research focused on early HC is scarce and mostly consists of small-scale retrospective studies. Moreover, comprehensive reviews specifically focusing on early HC are limited. Therefore, this review aims to summarize the significant literature and latest advancements in the diagnosis and treatment of early HC, with a special emphasis on endoscopic diagnosis and minimally invasive surgery.

## 2. Epidemiology

HC is relatively rare. The world age-standardized incidence rate of HC in 2022 was 0.89/100,000 (0.29 in women and 1.6 in men). HC accounted for 0.4% of all new cancer cases and 0.4% of all new cancer deaths in 2020.^[1]^ The global incidence rate of HC has increased over the past 3 decades and the mean age of individuals diagnosed with HC was approximately 65 years. Moreover, the disease is more prevalent in males than in females and more than 80 % are male, which is closely connected with the higher prevalence of smoking in men.^[3,13]^ The age-standardized incidence of HC exhibits regional variations, with the greatest prevalence in south-central Asia, followed by the territories of Western, Central and Eastern Europe, and the lowest incidences documented in North Africa and Western Asia. The differences in the distribution of incidence rates worldwide are most likely attributable to social customs related to tobacco consumption, alcohol intake and betel nut chewing. The risk factors of HC include tobacco, alcohol, betel quid chewing, oral healthconditions, human papillomavirus (HPV), bile reflux as well as Plummer-Vinson syndrome (PVS).

According to staging system of the American Joint Committee on Cancer 8th Edition, early HC generally includes stage I and stage II, namely T_1-2_N_0_M_0_. The diagnosis of early stage HC is relatively difficult, which often results in the disease already having progressed to an advanced stage by the time it is identified. Fewer than 1 in 4 patients suffering from HC are diagnosed in early stages.^[13]^ The unfavorable prognosis of HC primarily stems from the fact that hypopharyngeal malignancies frequently manifest in an advanced stage.^[3]^

## 3. Risk factor

Alcohol drinkers and smokers are at a markedly increased risk of HC than individuals who have never drunk or never smoked. More than 50% of patients with HC have a history of alcohol consumption. An independent and dose-responsive relationship exists between the incidence of HC and tobacco use as well as alcohol consumption.^[14]^

The International Agency for Research on Cancer has classified the areca nut as a carcinogen in Group 1 agent for humans. The associations of the areca nut with HC are less established in the current literatures. In a case-control study focusing on the population of south-central China, Zeng et al demonstrated that betel nut chewing was related to an increased incidence of hypopharyngeal cancer, identifying it as an important determinant.^[15]^

The relationship between suboptimal oral hygiene and a heightened risk of developing HNC has been established in studies across various geographical areas, such as China, Japan, the United States, Latin America, Central Europe and India.^[15,16]^ Indicators of suboptimal oral hygiene, including low frequency of tooth brushing, gum bleeding, tooth loss, the utilization of dentures, sparse dental consultations, and periodontal diseases, are considered potential contributors to the genesis of HNC. Maintaining good oral hygiene, evident through regular tooth brushing, minimal tooth loss, yearly dental consultations and absence of periodontal diseases correlates with a reduced risk of HNC.^[17]^ Zeng et al reported a link between poor oral hygiene and the rising incidence of HC, although supporting evidence from other studies remains limited.^[15]^ In addition, tumor microbiota is associated with tumor recurrence and metastasis among patients with HC.^[18]^ The oropharyngeal microbiota of HC patients showed dysbiosis with low diversity and abundance.^[19]^

The relationship between laryngopharyngeal reflux (LPR) with HC remains controversial. The main refluxed materials in LPR include gastric acid, pepsin, bile acids, bile salts and bacteria colonized in the gastrointestinal tract. Studies conducted both in vitro and in vivo have indicated which bile reflux is an independent risk factor for HC. The presence of acidic bile induces a continuous activation of NF-κB which can enhance the phenotype of carcinogenic miRNA and mRNA, promote the proliferation of mutant cells and result in the malignancy of hypopharyngeal mucosa with long-term exposure.^[20,21]^ Acidic bile also plays a carcinogenic role by promoting premalignant lesions with DNA damage and leading to abnormal activation of STAT3 that is considered an oncogene associated with the occurrence and development of HNC.^[20,21]^ Furthermore, the combination of bile acids and tobacco smoke nitrosamines may lead to a significantly increased risk of HC.^[22]^ In addition, prolonged exposure to pepsin reflux is recognized as a contributing factor towards the pathogenesis of HC.^[23]^

The infection of HPV plays an important role in elevating the risk of HNC, especially oropharyngeal squamous cell carcinoma.^[24]^ The etiological relation with the development of HC remains unestablished. HPV positivity rates in HC patients accounted for 15% to 25%.^[25,26]^ The infection of HPV correlates with HC prognoses, particularly yielding more favorable outcomes for patients with HPV-positive HC.^[27]^ The observed association might be attributed to the increased susceptibility to radiation observed in cancers associated with HPV.^[28]^

Characterized by a classic triad comprising iron-deficiency anemia, esophageal webs and dysphagia, PVS is an uncommon condition predominantly observed in white middle-aged females.^[29]^ There is a correlation between PVS and heightened risks of squamous cell carcinomas in the pharynx and esophagus, especially carcinogenesis of postcricoid carcinoma.^[30,31]^

## 4. Anatomy and pathology

Originating at the upper limit of the hyoid bone and descending to the cricoid cartilage’s inferior boundary, the hypopharynx is a mucosa-lined muscular tubular structure connecting the oropharynx, larynx, cervical esophagus. The hypopharynx serves as a passage for food and water to the esophagus as well as air to the lungs and plays an important role in speech, swallowing functions, and preventing aspiration. The hypopharynx’s surface is covered by stratified squamous epithelial tissue, organized in a sequence from inner to outer layers as follows: the mucosal layer, submucosa, muscularis propria, and adventitia. Notably, the mucosal stratum is devoid of muscularis mucosae, comprising solely of squamous epithelial cells and the underlying lamina propria. The hypopharynx is composed of the following 3 subsites: the pyriform sinus, the posterior pharyngeal wall, and the postcricoid area. There are abundant lymphatic drainage network linking hypopharynx to the deep cervical lymph nodes and the retropharyngeal lymph nodes. Innervation of the hypopharynx is provided by the pharyngeal plexus, which mainly includes the glossopharyngeal and vagus nerve branches. Motor functions of the hypopharyngeal constrictor muscles are governed by the vagus nerve, while sensation in the hypopharynx is transmitted by the glossopharyngeal nerve as well as internal branch of the superior laryngeal nerve originating from the vagus nerve. The blood supply of the hypopharynx primarily includes the superior pharyngeal artery as well as the superior and inferior thyroid arteries, which all stem from the external carotid artery.

Almost all HCs are epithelial in origin and hypopharyngeal squamous cell carcinoma (HPSCC) is the pathological type of over 95% of hypopharyngeal cases.^[13]^ Other rare histological types of HC include spindle cell squamous cell carcinoma, basaloid squamous cell carcinoma, carcinoma of the minor salivary glands, neuroendocrine carcinoma, hematolymphoid neoplasm, melanoma and soft tissue tumor. HC most frequently occurs in the piriform fossa area with approximately 50% of these carcinomas situated within the area. The posterior pharyngeal wall ranks next in frequency of occurrence, with the postcricoid area emerging as the least prevalent site.^[13,32]^ HC exhibits a significant aggressive profile with a marked risk of submucosal spread, perineural and vascular invasion as well as a propensity for early LNM and distant metastasis.^[4,33]^

## 5. Clinical presentation

Most patients with HC have no obvious symptoms and signs in the early stage until laryngeal invasion or cervical lymphadenopathy.^[34]^ Dysphagia, the most common clinical manifestation in HC cases, is progressively worsening and usually leads to significant malnutrition as well as weight loss. Other possible symptoms include hoarseness, neck mass, weight loss, sore throat, swallowing pain, globus hystericus, dyspnea.^[34]^

The cancer-specific survival and OS rates of patients with early cancers (stages I and II) are higher compared to those with locally advanced diseases (stages III and IVb) or distant metastasis (stage IVc),^[13]^ but HC is mostly found in the late stage. About 20 % of HC patients are already in stage III upon diagnosis. In excess of half of the cases with HC are in stage IV and 3% to 6% have distant metastasis at the initial presentation.^[13,34]^ The prevalence of LNM in cases of HC exceeds 70% with a marked correlation between LNM and the patient’s prognosis. The disease-specific survival (DSS) and OS rates for patients with HC involving LNM are considerably reduced in comparison to cases without LNM.^[32]^

The synchronous and metachronous second primary cancers (SPC) are one of the important causes of death in HNC.^[35,36]^ Second primary malignancies (SPM) are common with an incidence of 10% to 50% in patients with HC.^[37]^ Compared with other HNC, individuals with HC have a higher risk of SPC, typically in the lung, esophagus, head and neck.^[38,39]^ The most common type of SPM both in individuals with HC and in individuals with HNC is esophageal cancer.^[38–40]^ In cases of esophageal squamous cell carcinomas (ESCC), SPM are also more frequent in HNC, of which more than 60% are located in the hypopharynx.^[41]^ The mechanism of this phenomenon is not clear, but it can be explained by the “regional carcinogenesis theory.” The esophagus and the hypopharynx are anatomically adjacent with both mucosal surfaces being lined by squamous epithelium. After receiving the stimulation of common carcinogenic factors, patients with HC have an increased risk of SPM.^[42]^

## 6. Diagnosis

Early diagnosis and accurate staging are essential for the treatment of HC. Fiberoptic laryngoscopy combined with biopsy is helpful in distinguishing between benign and malignant lesions, as well as in identifying their histological subtypes. Contrast-enhanced computed tomography (CT) or magnetic resonance imaging (MRI) is utilized to evaluate primary lesions, local lymph nodes and cartilage invasion. MRI can provide better definition of soft tissue and has higher sensitivity for cartilage infiltration, which is used to assess the degree of cartilage infiltration and soft tissue involvement.^[9]^ Neck ultrasound is usually used for preliminary screening or guided puncture of lymph nodes. Positron emission tomography (PET)/CT scans can be used to detect distant metastasis and evaluate prognosis of HC.^[10,33]^ Additionally, the National Comprehensive Cancer Network (NCCN) clinical practice guidelines for HC recommend conducting multidisciplinary assessments based on clinical symptoms, including nutritional assessment, speech and swallowing assessment, as well as dental assessment.^[12]^

HC often receives a diagnosis at an advanced phase and may require extensive surgical resection, which may lead to the impairment of swallowing and speech function, thereby greatly diminishing the patients’ quality of life. While the prognosis of early HC is better, early diagnosis of HC is difficult. Tumors in the pyriform sinus are difficult to detect by routine fiberoptic endoscope because the hypopharynx is a narrow space that limits the visual field of conventional endoscopy. Since the hypopharynx is a highly wrinkled lacunar organ that makes CT unclear on some of the hypopharyngeal anatomical substructures, hypopharyngeal neoplasms may be missed in conventional imaging.^[43]^ MRI may not detect early lesions and over-evaluate tumor volume due to the limitation of spatial resolution.^[44]^ Lugol chromoendoscopy generally can't be used to the pharynx because of the high risk of aspiration and severe mucosal irritation.^[8]^ Advances in endoscopic techniques, including ME-NBI, have made it possible to detect superficial HC.^[8,43,45–47]^

### 6.1. Narrow-band imaging

NBI system is an advanced optical imaging technology. The working principle of NBI is to use NBI filters to filter white light into lights of distinct wavelengths, namely, the blue and green lights with center wavelengths of 415 nm and 540 nm, correspondingly. These specific center wavelengths correspond to the high absorption peaks of hemoglobin, leading to their absorption by hemoglobin and only penetration through the surface layers of human tissue. The blood vessels under NBI show dark color, which enhances the contrast of mucosal surface structure and makes blood vessels and other tissues on the mucosa surface more visible. The capillaries on the mucosal surface and the veins in the submucosa are shown as brown and cyan, respectively.^[8,46,48]^ The magnifying endoscope (ME) has the ability of image magnification, thus the microvessel morphology on the mucosal surface can be more clearly and brightly seen by NBI combined with ME.

ME-NBI has been utilized for the detection of neoplasm in esophagus, colon, rectum, bladder, head and neck.^[49–51]^ A clearly defined brown area, along with irregular microvascular structures observed by ME-NBI, is used as an effective marker for superficial pharyngeal cancers. NBI improved the detection rate of superficial pharyngeal cancers and is superior to traditional white light endoscopy.^[8,47,49,52–54]^ Studies have shown that NBI is more effective than white light imaging,^[55]^ Lugol chromoendoscopy^[56]^ and PET/CT^[57]^ in detecting SPC of patients with HNC.

**Figure 1.**
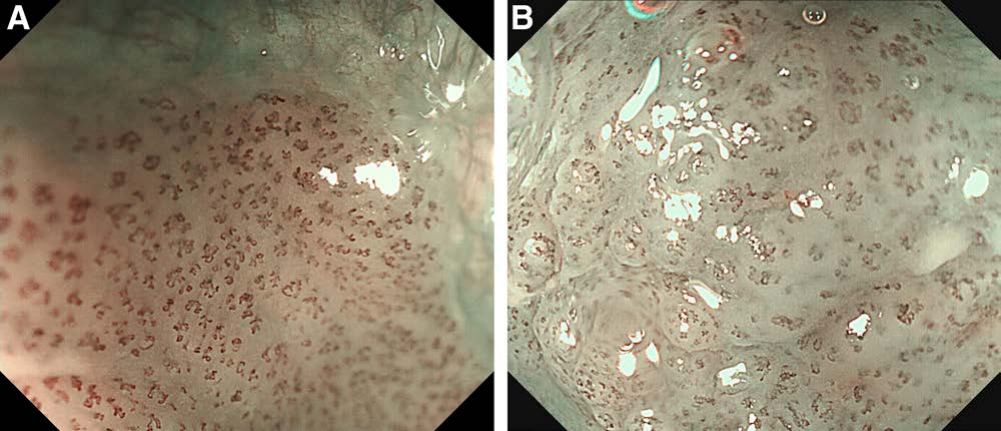
(A and B) Type B1, Type B vessel with a loop-like formation (caliber approximately 20 μm).

**Figure 2.**
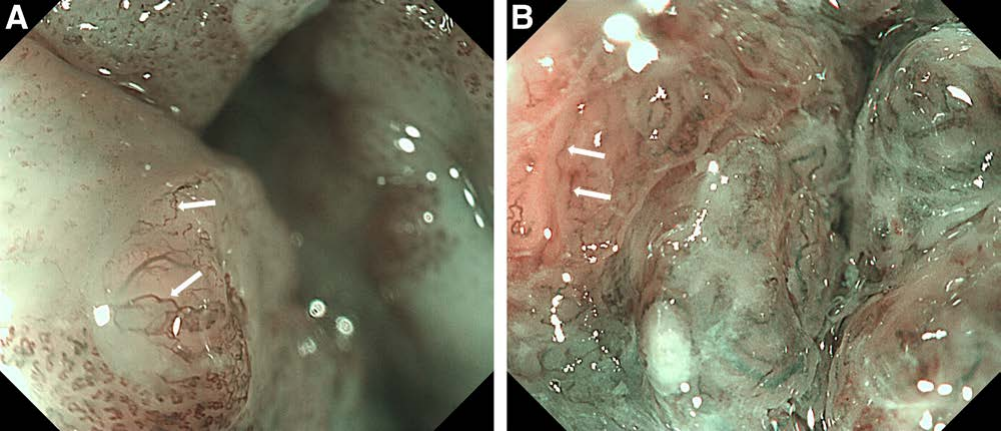
(A and B) Type B2, Type B vessel without loop‐like formation (white arrowhead).

**Figure 3.**
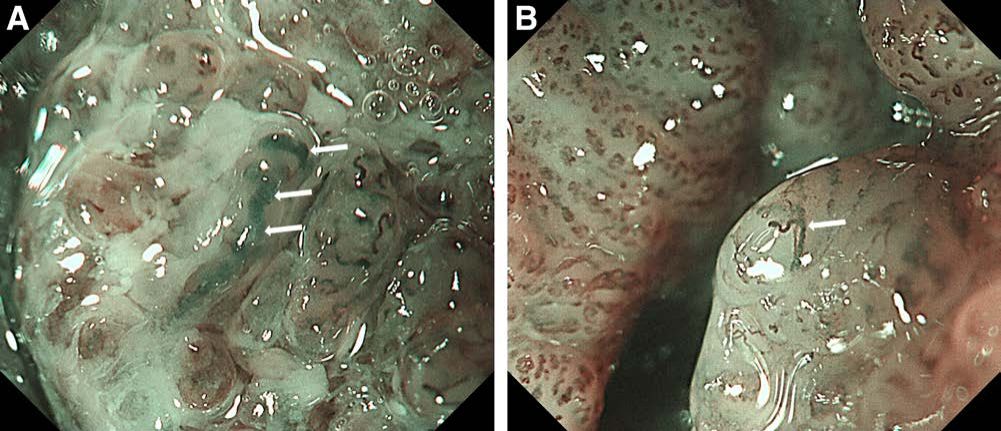
(A and B) Type B3, highly dilated Type B vessels with a caliber more than 3 times that of Type B2 vessels (caliber often > 60 μm).

ME-NBI is capable of assessing the invasion depth in superficial pharyngeal cancers by observing the microvascular patterns of the tumor surface.^[58,59]^ The microvascular pattern observed by ME-NBI has a close association with the invasion depth of superficial ESCC. The committee of the Japan Esophageal Society (JES) developed a simplified microvascular classification in order to predict the invasion depth of superficial ESCC.^[60,61]^ Microvessels are classified into Type A and Type B based on the degree of irregularity of microvascular morphology. Microvascular morphological factors include different shape (i.e., shape non-uniformity), irregular caliber (i.e., caliber variation), dilatation (i.e., enlargement or expansion) and weaving (i.e., tortuosity). Type A vessels refer to normal intra papillary capillary loops or mild atypia of intra papillary capillary loops. Type B vessels are abnormal microvessels characterized by severe irregularity or marked dilatation. Type B vessels are divided into 3 subtypes (B1, B2 and B3) based on the expansion degree and running patterns. Type B1 (Fig. 1A and B), B2 (Fig. 2A and B), and B3 (Fig. 3A and B) microvessels are respectively constituted by Type B microvessels with loop‐like formations, Type B microvessels lacking loop‐like formations, and highly dilated Type B microvessels whose calibers exceed 3 times that of Type B2 microvessels (often surpassing 60 μm). Only Type B1 found in the target lesions is diagnosed as tumors confined within mucosal epithelium (T1a-EP) or as tumors invading into lamina propria mucosa (T1a-LPM). Type B2 vessels found anywhere in lesions are predicted as tumors invading lamina muscularis mucosa (T1a-MM). Type B3 observed anywhere in target lesions indicates tumors invading into the middle third of the submucosal layer (T1b-SM2) or deeper.

**Figure 4.**
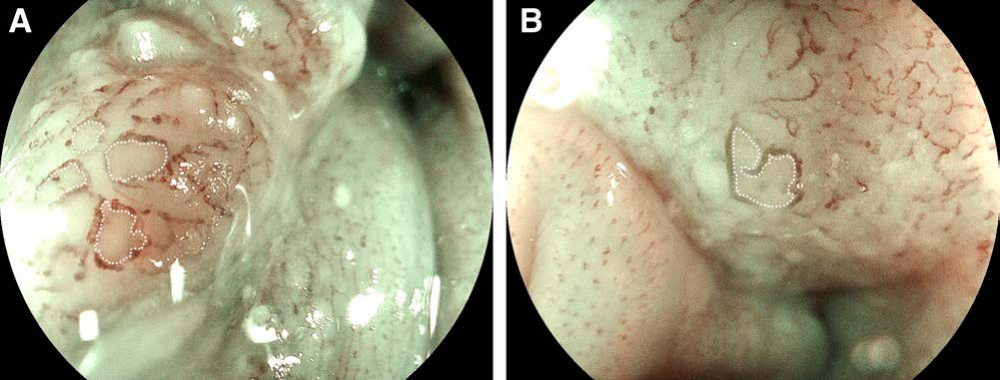
(A and B) Avascular area (AVA), a poor or no vascular-free region surrounded by Type B vessels.

In addition, avascular area (AVA) (Fig. 4A and B) is described as a poor or no vascular region enclosed by Type B vessels.^[60,61]^ AVA is classified into 3 types according to size in diameter: AVA-small (<0.5 mm), AVA-middle (0.5 mm or between 0.5 and 3 mm) and AVA-large (3 mm or larger). Any types of AVA encircled by only Type B1 vessels suggest the invasion depth as T1a-EP or T1a-LPM. The small, middle, and large AVAs encircled by vessels of Type B2 or B3 indicate the invasion depths as T1a-EP or LPM, T1a-MM or T1b-SM1, and T1b-SM2, respectively.

Some studies have shown that JES classification can also be applied to predict the invasion depth of superficial HC.^[58,59,62]^ Superficial HCs are limited to the epithelial and submucosal layers and do not penetrate deep into the muscular layer. Owing to the absence of muscularis mucosa in the hypopharynx, the application of JES classification in superficial HC is different from that in superficial ESCC. Only Type B1 vessels found in the target lesions indicate HC in situ (Tis), whereas Type B2 and Type B3 vessels found in any location of target lesions are predicted as subepithelial invasion.^[58,59,62]^ AVA-small can be used to predict Tis. AVA-middle and AVA-large can be used to predict submucosal infiltration. Yamaguchi et al reported that Type B2/B3 microvessels and AVA-middle/large were independent predictors of subepithelial invasion. The tumor thickness and the LNM rates in pharyngeal cancers gradually increased in the order of B1 to B2 to B3.^[62]^ Although these studies have shown that the JES classification was helpful in determining the invasion depths of superficial HC, these studies are mostly single-center retrospective studies and are focused on the Asian region. Therefore, further validation is needed through multicenter prospective studies and research in other regions.

### 6.2. Artificial intelligence-assisted diagnosis

Artificial intelligence (AI) has shown great potential across various medical fields in recent years. AI can extract and learn from massive data in a short time for disease screening, detection, diagnosis, treatment and prognosis prediction.^[63–65]^ AI mainly includes deep learning (DL) and machine learning. The convolutional neural network (CNN) is one of the DL algorithms, which contributes to automatically extract and detect imaging features from medical images via multiple stacked convolutional layers for lesion segmentation, recognition, detection and medical image classification.^[66]^

At present, there have been some studies applying AI to the diagnosis, treatment and prognosis of pharyngeal cancers. Nakajo et al found that the AI model has high diagnostic accuracy in the anatomical location classification of endoscopic images of the pharynx and larynx. The weighted average precision, recall, and F1-score of the AI model were 0.934, 0.933, and 0.933, respectively, supporting its effectiveness in precise anatomical classification tasks.^[67]^ Tamashiro et al created a diagnostic system for detecting pharyngeal cancers from static endoscopic images based on CNN, which detected pharyngeal cancers in images obtained via NBI with a sensitivity of 85.6% and via white light with a sensitivity of 70.1%.^[68]^ Kono et al developed a diagnostic system for pharyngeal cancer utilizing Mask region-based CNN and verified that the AI system presented a promising prospect, with high sensitivity of 92% and specificity of 47% in an evaluation utilizing endoscopic video imagery for real-time diagnostic simulation.^[69]^ Li et al introduced a laryngopharynx AI diagnosis system grounded in deep CNNs, with a high level of stability and accuracy in the real-time automatic identification of LPC in both white light imaging and NBI images. Across all external datasets, the system achieved sensitivity, specificity, and negative predictive value >90%, with area under the curve values ranging from 0.965 to 0.987.^[70]^ Although there have been some studies on exploring AI for the diagnosis of HC, the majority of these studies are retrospective and research targeting the specific application of AI in diagnosing and treating superficial HC remain scarce.

There are several limitations and hurdles when applying AI within the realm of medicine.^[64,65,71,72]^ DL is currently criticized as a “black box” for its opacity. It is difficult for researchers to understand data analysis and decision-making mechanisms within DL models as these models involve a large number of parameters.^[72]^ Systems whose decisions cannot be well explained are difficult to be trusted. There have been many studies on interpretable machine learning methods, most of which refer to image classification and generate saliency maps that highlight the influence of different image regions.^[71]^ Due to the variations of clinical medical data across different regions or institutions, the efficacy of DL models often decreases when used in different healthcare institutions.^[73]^ Additionally, the inherent biases in the datasets used for training and testing AI models can result in biased decision-making, favoring certain racial and ethnic groups. These biases can inadvertently be built into healthcare algorithms.^[64]^ Data sharing and code sharing of AI models can help address this issue. Due to the substantial requirement of medical data for AI training, privacy protection and the security of healthcare data are inevitably involved and must be carefully considered.

## 7. Treatment

Patients with early HC are recommended to undergo a single-treatment modality, either conservative surgery or definitive radiotherapy. The surgical approaches of early HC include traditional open conservation surgery and transoral minimally invasive surgery. Compared to traditional open surgery, patients with early hypopharyngeal cancer who undergo either radiotherapy or transoral minimally invasive surgery can achieve better oncologic outcomes. Given the high incidence of SPM in this population, which negatively affects OS, transoral minimally invasive surgery is often preferred as the initial treatment modality. This strategy preserves the option of reserving radiotherapy or chemoradiotherapy (CRT) for the management of potential future second primary tumors.^[9,74–76]^ In cases of early stage HC with occult cervical LNM, in addition to the primary lesion excision, cervical lymph node dissection in zones II to IV on the ipsilateral or bilateral sides is required.

Patients with locally advanced HC should receive multimodal treatment due to LNM.^[2]^ Several studies have indicated that, in cases of locally advanced and advanced hypopharyngeal cancer, surgical treatment andCRT offer similar survival outcomes.^[77,78]^ For patients with T1-2, N1-3, M0 and T3, N0-3, M0 HC, those who initially receive primary surgical treatment including partial or total laryngopharyngectomy should undergo postoperative radiotherapy or CRT according to pathologic features, while those initially receiving nonsurgical treatment should undergo concurrentCRT or induction chemotherapy.^[10]^ For patients with T4, N1-3, M0 HC, T4a patients suitable for surgery require total laryngopharyngectomy and neck dissection (ND), typically accompanied by adjuvant radiotherapy, induction chemotherapy or CRT. For T4 patients unfit for surgery, the main alternative treatment includes induction chemotherapy, concurrent CRT or radiotherapy alone, only in those who are ineligible for CRT and refuse total laryngectomy (TL). For the management of recurrent and/or metastatic diseases, multidisciplinary evaluation is crucial for establishing the optimal treatment strategy.^[9,10,12]^ Palliative treatment includes radiotherapy, surgery, analgesia, and other treatment modalities to control symptoms of disease spread and enhance quality of life for individuals with very advanced HC. For patients with regional and/or local carcinoma recurrence without distant metastatic disease, re-irradiation or salvage surgery along with systemic treatments can be used in select cases. In the absence of distant metastasis, surgery for resectable recurrent or persistent locoregional disease is recommended. Individuals with metastatic disease or locoregional recurrence ineligible for local salvage treatments should undergo systemic therapy. Locoregional treatment may be fit for individuals with oligometastatic or a single metastatic lesion, such as surgery, radiotherapy, ablation therapy. Immunotherapy may be considered for all patients with distant metastatic, unresectable, or recurrent lesions unsuitable for radiotherapy or surgery.^[12]^

### 7.1. Traditional open surgery

Patients with superficial HC typically undergo conservative surgical treatments, in addition to emphasizing organ preservation and functional outcomes. Some studies have indicated that open conservative approaches achieved comparable oncological outcomes to those achieved through radical surgical approaches, offering enhanced survival rates and functional preservation.^[79,80]^ Previous evidence has shown that the 5-year overall survival rate for patients with HC undergoing partial pharyngectomy ranges from 50% to 70%.

The degree of preservation of larynx and hypopharynx functions depends on aggressiveness of the primary tumors, size and location. Therefore, the selection of a surgical method and the extent of surgical resection should be established in accordance with the patient’s specific situation. For lesions located in the lateral wall and the posterior wall of the hypopharynx, partial hypopharyngectomy can be considered through a lateral pharyngeal wall incision or via subhyoid pharyngotomy.^[81,82]^ However, lesions involving the apex of the piriform sinus or the postcricoid area are not recommended to undergo this procedure. For tumors located in the aryepiglottic fold and the piriform sinus, partial laryngopharyngectomy is feasible but not advised for lesions with involvement of vocal cord fixation, the cricoid cartilage, the thyroid cartilage and the apex of the piriform sinus.^[80,83]^

Supraglottic hemilaryngopharyngectomy, combined with ipsilateral ND for lesions in the upper medial piriform fossa and aryepiglottic folds, offers satisfactory functional outcomes in some retrospective studies.^[79,84]^ The technique of supracricoid hemilaryngopharyngectomy, introduced by Laccourreye et al^[85]^ in 1965, targets limited tumors on the piriform sinus’s lateral wall and preserves physiological functions such as voicing, breathing and swallowing while achieving local control rates of tumors comparable to those of TL.^[86]^ Vertical hemilaryngopharyngectomy is initially applied for small tumors in the lateral wall of the piriform sinus, and indications were later extended to tumors originating in the piriform sinus that spread to the cricoid cartilage, the thyroid cartilage and the apex of the piriform sinus.^[87]^

The majority of patients encounter challenges related to defect reconstruction after HC resection. Reconstruction surgery after HC resection focuses on balancing tumor eradication with speech preservation. The purpose of reconstruction is to ensure the preservation of swallowing function and laryngeal function and to reduce the incidences of fistula and stenosis.^[88,89]^ The selection of a reconstruction strategy is mainly contingent upon the extent of the defect created during lesion resection.^[82]^ After TL combined with partial pharyngectomy, direct closure of the pharynx defect is accomplished using the preserved pharyngeal mucosa wherever possible. When direct closure of the pharyngeal defect is not feasible due to insufficient width of the remaining pharyngeal wall, latissimus dorsi myocutaneous flaps, the supraclavicular artery island flap and pectoralis major flap may be utilized to repair the pharyngeal defect.^[90]^ In cases of tumors extensively involving the posterior pharyngeal wall or cervical esophagus, total laryngopharyngectomy results in circumferential pharyngeal defects posing significant challenges for reconstruction. Pectoralis major myocutaneous flaps, jejunal free flaps, fasciocutaneous free flaps (anterolateral thigh or radial forearm flaps) are options for repairing these extensive defects.^[91]^ For patients requiring total laryngopharyngectomy and esophagectomy due to extensive esophageal tumor infiltration, gastric pull-up (pharyngogastric anastomosis) and colon interposition are employed for reconstruction.^[89]^

Traditional open surgeries, especially total laryngopharyngectomy, involving extensive pharyngeal and laryngeal resections are associated with the impairments of swallowing and/or speaking functions, cosmetic deformities and extended hospital stays.

### 7.2. Transoral minimally invasive surgery

Patients with advanced HC traditionally undergo treatments including open surgical resection, radiotherapy, or chemotherapy. These treatments may result in complications including xerostomia, cosmetic deformity, dysphagia, thickened saliva, and vocal cord damage, which significantly deteriorate the quality of life in individuals with HC. With the progression of endoscopic technologies, NBI and ME have notably improved the detection rates of early stage lesions. The adoption of minimally invasive techniques not only reduces tissue damage but also preserves the pharyngeal and laryngeal functions, subsequently improving the patients’ quality of life.

Transoral surgeries for HC encompass several techniques, such as transoral laser microsurgery (TLM), transoral robotic surgery (TORS), endoscopic laryngopharyngeal surgery (ELPS), endoscopic mucosal resection (EMR) and endoscopic submucosal dissection (ESD).

#### 7.2.1. Transoral laser microsurgery

TLM was first proposed for treating laryngeal cancers by Strong and Jako, and was later developed and extended to HC by Steiner. This technique utilizes a carbon dioxide (CO2) laser to precisely excise tumor tissue. Indications for TLM include early stage HC (such as T1 and select T2 cases) as well as selective cases of advanced HC. TLM offers survival rates comparable to traditional open surgery, but with significantly reduced surgical trauma, shorter postoperative recovery time, lower risk of complications, and better preservation of phonation and swallowing functions. Given the high propensity for LNM in HC, unilateral or bilateral ND is recommended concurrently with TLM.

In a prospective case-series study including 172 patients with HC, 150 of whom had pyriform sinus tumors eligible for the criteria for TLM resection, Martin and Steiner et al^[92]^ reported that the study covered lesions from T1 to T4 and all patients underwent piecemeal resection by TLM with 93% of patients undergoing ND and 52% receiving adjuvant radiotherapy. The local control rates for T1 and T2 at the 5-year mark were 84% and 70%, respectively. The recurrence-free survival rate for stages I and II at the 5-year mark was 73%. Patients with HC treated with TLM experienced a high rate of larynx preservation and a low rate of complications, highlighting TLM’s efficacy in managing early stage HC. In another retrospective single-center study of 211 individuals with HC, Weiss et al^[93]^ found that TLM achieved 5-year local control rates of 88.1% for T1 tumors and 74.8% for T2 tumors. The 5-year OS rate for stages I and II was 68.2%. Furthermore, 4% of patients required a permanent gastrostomy. These studies collectively suggest that incorporating TLM as a primary treatment within multimodal treatments yields favorable oncologic outcomes and high preservation rates of organs and their functions.

The limitations of TLM include technical complexity and restricted visibility of the surgical field.^[94,95]^ These constraints necessitate a high level of surgical expertise and extensive experience. Furthermore, TLM may not be the optimal option for tumors with extensive infiltration or a wider base, as these factors may limit the feasibility of complete tumor resections.

#### 7.2.2. Transoral robotic surgery

TORS, introduced by Weinstein and O’Malley in America in 2005 and progressively established for utility in oropharyngeal cancer, offers a clear surgical field and flexible operational capability by integrating robotic systems with endoscopy. TORS initially achieved favorable outcomes in the management of oropharyngeal cancer and has gradually extended its applications to hypopharyngeal and laryngeal cancers.

In the field of HC treatment, TORS is still in the early stage of clinical application. However, small-scale studies and selected case reports have demonstrated its safety and efficacy in treating localized T1-T2 HC.^[96]^ TORS offers high surgical precision with the ability to filter out the surgeon’s hand tremors, minimizing damage to surrounding tissue while excising the tumor. This technique not only reduces the need for tracheotomy and reconstructive surgeries but also is characterized by minimal intraoperative bleeding, shorter operation times, fewer postoperative complications, and quicker recovery of swallowing function compared with open surgical procedures.^[94]^ Mazerolle et al^[96]^ applied TORS to 57 individuals with HC, with an overwhelming 98% being in T1-T2. The disease-free survival and OS rates were 74% and 84% at 2 years, respectively, while these rates decreased to 50% and 66% at 4 years, respectively. The organ preservation rate was 96% by the end of the follow-up. The primary surgical complication reported was hemorrhage. In a systematic review concerning TORS and TLM, De Virgilio et al reported that among 105 patients treated with TORS, the cumulative survival rate was 85.5%, with laryngeal function preservation observed in 95 cases.^[97]^ TORS offers a promising alternative treatment option for HC, in conjunction with simultaneous or isolated ND.

There are some limitations in the application of TORS in HC. The primary limitation is the significant cost associated with the robotic system,^[95]^ in addition to challenges such as restricted visibility of the surgical field and the potential for instrumental interference within the confined spaces of the hypopharynx.

#### 7.2.3. Endoscopic laryngopharyngeal surgery

ELPS, similar to ESD and developed from ESD, is primarily performed by an otolaryngologist with the assistance of a gastroenterologist’s endoscopy.^[98]^ ELPS is distinguished by its superior postoperative swallowing function, satisfactory oncologic outcomes and limited complications compared to traditional open surgery.

Using a curved laryngopharyngoscope, ELPS provides an exceptional view of the hypopharynx, allowing for enhanced lesion exposure and precise surgical manipulation. In contrast to EMR, which may require piecemeal resection for extensive or protruding lesions, ELPS allows for en bloc resection. Furthermore, ELPS can be applied to select cases of HC with partial esophageal invasion, employing combined therapy with ESD.^[99,100]^ The main complications associated with ELPS are postoperative bleeding and subcutaneous emphysema. In a clinical study involving 118 patients undergoing ELPS, Kishimoto et al reported a 5-year OS rate of 85.5% and a 3-year OS rate of 93.6% with clear visibility of hypopharyngeal area during the procedure.^[98]^ They found that ELPS enables tumor resection with narrow surgical margins (approximately 1–2 mm), while still achieving satisfactory oncological outcomes. In another retrospective study of 258 individuals with superficial pharyngeal cancers treated with ELPS, Akihito et al^[100]^ observed 3-year cause-specific survival and OS rates of 100% and 85.7%, respectively, with no cases of vocal cord paralysis and minimal complications. Because of the high rate of LNM in HC, ND should be performed when cervical LNM is present.

The application of ELPS is confined to specific indications, mainly targeting superficial carcinomas like in situ or early stage cancer. The limitations of ELPS include the instruments interference within the operational space and challenges in accessing specific regions, notably the tumor’s anal aspect and areas near the esophageal entrance.^[99]^

#### 7.2.4. Endoscopic mucosal resection

Endoscopic approaches for superficial HC have evolved over time, with EMR being the earliest technique introduced, followed by the adoption of ESD, and more recently, ELPS has begun to be applied. EMR is less invasive and beneficial for the resection of small superficial lesions. Compared to ESD, EMR offers shorter operation times, quicker recovery periods and lower risk of complications. In a single-center retrospective study of 31 patients with 37 pharyngeal lesions treated with EMR, Suzuki et al^[101]^ reported a median operation time of 45 minutes, a median hospital stay of 7 days, and no recurrence or metachronous tumors in 20 patients by the end of the follow-up period.

There are some limitations of EMR including difficulties in achieving complete resection for larger or more complex lesions, which may lead to higher recurrence rates compared to more invasive methods. The higher local recurrence rate of EMR compared to ESD may be attributed to the fixed and complex anatomical structure of the pharynx, making it challenging to achieve en bloc resection through EMR.

#### 7.2.5. Endoscopic submucosal dissection

ESD represents a significant advancement beyond EMR, as the latter technique has limitations in effectively treating lesions of larger size and depth. ESD, developed in Japan in the late 1990s, revolutionized the management of early stage gastrointestinal cancers by enabling en bloc resection of bigger and deeper tumors. This approach facilitates precise histopathological assessment, essential for staging and further treatment planning. Moreover, ESD’s minimally invasive characteristic leads to shorter hospital stays and quicker recovery times, greatly benefiting patient care. ESD has been applied in a variety of diseases, primarily focusing on the management of early carcinomas and precancerous lesions in the digestive tract.^[102]^ Furthermore, recent advancements are also exploring the use of ESD in early stage lung and bronchial tumors, superficial bladder tumors as well as nasopharyngeal and hypopharyngeal lesions.

The detection of superficial hypopharyngeal cancer using NBI has enabled endoscopists to perform curative resections via ESD or EMR. Several studies have demonstrated that ESD is an effective and safe minimally invasive approach for early HC and precancerous lesions.^[103,104]^. Satake et al evaluated 50 lesions of superficial pharyngeal squamous cell carcinoma with subepithelial invasion in 47 patients treated with EMR-C, ESD, or ELPS. They reported a local recurrence rate of 13%, and 5-year OS and DSS rates of 84.5% and 100%, respectively.^[105]^ In a prospective study involving 54 patients with 73 lesions of superficial pharyngeal cancer, ESD achieved a complete resection rate of 79.5%, an en bloc resection rate of 100%, a recurrence-free survival rate of 98.1%, and a 3-year OS rate of 97.7%, without any severe adverse events caused by ESD.^[106]^ In a systematic review of 10 studies on ESD for the treatment of superficial pharyngeal cancers, Kamal et al also concluded that ESD was a safe and effective treatment for superficial pharyngeal cancers, as evidenced by a high en bloc resection rate of 94%, a complete resection rate of 72%, a low adverse event rate of 10%, and a local recurrence rate of just 1.9%.^[95]^

ESD for HC provides higher en bloc resection rates than that of EMR, albeit with longer operation times and elevated risks of complications.^[107–110]^ A comparative study of ESD and EMR for early mesopharyngeal cancers and HC demonstrated the clinical superiority of ESD, with significantly higher en bloc and R0 resection rates (98% and 79%, respectively) compared to EMR (37% and 26%). However, ESD was associated with a slightly higher risk of complications, such as subcutaneous emphysema, while no adverse events occurred in the EMR group.^[107]^ In another retrospective analysis of 45 cases of superficial pharyngeal cancer from Japan, Hanaoka et al 107 found that ESD achieved higher en bloc resection and curative resection rates (100% and 81.6%, respectively) than EMR (59 and 50%, respectively).^[108]^ Okada et al analyzed 76 superficial pharyngeal carcinomas in 59 consecutively treated patients, comparing ESD (n = 31 lesions) and conventional EMR (n = 45 lesions). ESD achieved higher complete and en bloc resection rates (54.8% and 77.4%, respectively) than EMR (28.9% and 37.8%, respectively).^[109]^ ESD proved to be more efficacious than EMR in the resection of HC. In addition, the results from a single-center retrospective study indicated that ESD combined with ELSP was more effective in the resection of HC, with faster procedure speed and higher R0 resection rate than ELSP alone.^[99,100]^

### 7.3. Nonsurgical Treatment

Radiotherapy alone emerges as another viable treatment modality for T1-2, N0 HC patients apart from surgical interventions.^[9]^ When integrated with systemic therapy, it further contributes to the curative treatments of locally advanced carcinomas. The employment of postoperative radiotherapy or CRT is beneficial for patients with high-risk factors such as positive surgical margins or extracapsular nodal spread, improving OS and local-regional disease control rates. Considering the greater risk of local or locoregional recurrence over distant metastases, cancers that are demonstrated resistance to radiation may often be amenable to surgical rescue. A multicenter study focusing on early stage HC treated with radical radiotherapy documented a 5-year OS rate of 66.0% and a DSS rate of 77.4%. Moreover, 56.5% of these cases presented with synchronous or metachronous SPC, and 10 individuals succumbed to SPC.^[5]^ Yoshimura et al^[111]^ observed that among 77 patients treated with radical radiotherapy, the 5-year OS, recurrence-free survival, DSS, and laryngeal preservation rates were at 47%, 57%, 74% and 70%, respectively. Additionally, a considerable percentage (83%) were diagnosed with either synchronous or metachronous cancers. These findings underscore the effectiveness of radical radiotherapy for the treatment of early stage HC, albeit the relatively high incidence of concurrent or subsequent malignancies may potential impact on survival outcomes.

Chemotherapy is mostly used for HC at locally advanced or very advanced stages. The most effective application of induction chemotherapy in HC remains a contentious issue. Due to the presence of LNM, patients with locally advanced HC often necessitate comprehensive treatment modalities. For the majority of individuals with T1-3, N1-3 stage HC, primary treatment typically involves cisplatin-based CRT, positioning surgery as a secondary option for salvage therapy.^[4]^ Concurrent CRT can increase OS and locoregional control rates in comparison to radiotherapy alone. Chemotherapy has a good utility among organ preservation methods, with survival rates comparable to surgery.^[112]^ Induction chemotherapy can decrease the incidence of distant metastases while its impact on OS is inconsistent.^[9]^

## 8. Conclusion

HC remains one of the malignancies with the poorest prognosis among HNC. The treatment of locally advanced and very advanced HC is still controversial, necessitating the collaboration of multidisciplinary teams to develop comprehensive multimodal treatment plans. Immunotherapy and molecular targeted therapy represent promising future directions. Patients with early HC who undergo conservative surgery or radiotherapy alone achieve similar oncologic outcomes and functional preservation. The advent of ME-NBI and AI-assisted diagnosis has inaugurated a new epoch of early detection for such malignancies, laying the foundation for the expanded adoption of minimally invasive techniques, especially endoscopic surgery. The future treatment directions for early HC include improved radiation therapy and innovative developments in minimally invasive surgery to further improve survival rates and preserve laryngopharyngeal function.

## Acknowledgments

We thank all staff who devoted their time and efforts to the study.

## Author contributions

**Conceptualization:** Xiaowei Tang, Yu Bao.

**Resources:** Rui Zhao.

**Supervision:** Xiaowei Tang.

**Writing – original draft:** Jinhao Cheng, Yuzhi Liu.

**Writing – review & editing:** Xiaowei Tang, Rui Zhao.
